# *M. tuberculosis* in Lymph Node Biopsy Paraffin-Embedded Sections

**DOI:** 10.1155/2011/127817

**Published:** 2011-12-13

**Authors:** Abdurehman Eshete, Ahmed Zeyinudin, Solomon Ali, Solomon Abera, Mona Mohammed

**Affiliations:** Department of Medical Laboratory Sciences and Pathology, College of Public Health and Medical Sciences, Jimma University, 409 Jimma, Ethiopia

## Abstract

*Background*. Tuberculosis lymphadenitis is one of the most common forms of all extrapulmonary tuberculosis. *Objective*. To evaluate the magnitude of *M. tuberculosis* from lymph node biopsy paraffin-embedded sections among suspected patients visiting the Jimma University Specialized Hospital. *Method*. A cross-sectional study design of histological examination among lymph node biopsy paraffin-embedded sections by Ziehl-Neelsen and hematoxylin/eosin staining technique was conducted from December, 2009, to October, 2010, at the Department of Medical Laboratory Science and Pathology. *Result*. Histopathological examination of the specimens by hematoxylin and eosin staining technique revealed the presence of granulomas. But for the caseation and necrosis they were present in 85% cases of nodal tissue biopsies. From those, 56.7% were from females. The presence of acid-fast bacilli was microscopically confirmed by ZN staining in 37 (61.7%) of the nodal tissue biopsies. *Conclusion and Recommendation*. Tuberculosis lymphadenitis is significantly more common in females. Hence, attention should be given for control and prevention of extrapulmonary tuberculosis.

## 1. Introduction

Tuberculosis (TB) remains a major global public health problem. It is estimated that about one-third of the world's population is infected with *Mycobacterium tuberculosis. *There were an estimated 8 million new cases of TB, resulting in 1.9 million deaths, with the greatest burden of disease in developing nations [1–3].

The genus *Mycobacterium* consists of nonmotile, non-spore-forming aerobic acid-fast bacilli. The cell wall is lipophilic and resistant to many disinfectants as well as to common laboratory stains. Currently, more than 70 species of *Mycobacterium* have been identified, many of which are associated with human diseases [4].

A group of *Mycobacterium* species called *M. tuberculosis* complex, comprise *M. tuberculosis, M. bovis*, and *M. africanum,* is of at most clinical importance since it causes tuberculosis in humans worldwide. Mycobacteria species other than those of the tuberculosis complex, also called nonmycobacteria, are widely distributed in the environment and may colonize and occasionally cause infections in humans [5].

The incidence of tuberculosis has stabilized or declined in most of the world WHO regions. On the other hand, it is increasing in Africa, Southeast Asia, and the Eastern Mediterranean. This is associated with high prevalence of the pandemic human immunodeficiency virus (HIV) in these regions. More than 4,000 people died daily in both pulmonary and extrapulmonary tuberculosis-related illnesses [6].

Tuberculosis infection can be of pulmonary or extrapulmonary type. After primary infection, TB may reactivate at anytime and anywhere in the body. Recent studies have suggested that the sites of extrapulmonary tuberculosis are lymph nodes in the neck, the bones, the serous membranes, and the cervical region. Tuberculosis of the lymphatic system is one of the most common of all extrapulmonary tuberculosis [1, 7, 8].

Lymph nodes involvement is the most common form of extrapulmonary tuberculosis. The synergy between tuberculosis and HIV infection and other immune-compromising conditions have resulted in an increase in the incidence of tuberculosis lymphadenitis and have further complicated tuberculosis control. HIV-related extrapulmonary tuberculosis is a World Health Organization (WHO) clinical stage four diagnosis, and patients with HIV-related extrapulmonary tuberculosis often have disseminated disease and are at high risk of rapid clinical deterioration and death [9–11].

Tuberculosis lymphadenitis occurs relatively early after primary infection with *M. tuberculosis* and often affects young people in countries with a high prevalence of tuberculosis. In children, the most serious forms are disseminated tuberculosis and tuberculosis meningitis. Tuberculosis lymphadenitis is the most common form, accounting for up to 50 percent of extrapulmonary cases in children [12, 13].

The extrapulmonary variety is now beginning to emerge from the shadows of pulmonary tuberculosis. In countries with good surveillance data like USA where the rate of pulmonary tuberculosis has declined to its lowest levels ever in 2001, statistics indicate a relative increase of extrapulmonary cases from 16% in 1992 to 20% in 2000. In USA the prevalence of extrapulmonary among immune competent persons was 15–30% [14].

Extrapulmonary tuberculosis (EPTB) has existed as a disease entity for centuries. It is a milder form of disease in terms of infectivity as compared to pulmonary tuberculosis. In India, EPTB comprises 20% of all TB cases. Its prevalence in the country varies between 8.3 and 13.1% in different districts in 2002. In the year 2006, 183,180 EPTB cases were registered in comparison to 555,660 smear pulmonary TB cases giving a ratio of 1 : 0.3. Cure of infectious cases is likely to have resulted in a relative rise of annual EPTB case detection. Prevalence of EPTB has also been found to be higher in paediatric cases [7, 15].

Extrapulmonary tuberculosis can occur alone or in combination with the pulmonary variety. It is usually confined to a single site, but disseminated form may also occur. Many of the affected sites may require an invasive procedure to get a biological sample to reach a diagnosis. Extrapulmonary tuberculosis remains a diagnosis that is often difficult to establish immediately and available [16].

## 2. Method and Materials

First ethical clearance was obtained from the Jimma University ethical review board. The purpose of the study was clearly explained for the study subjects, and written consent was obtained from each study participants. Confidentiality of the result was kept by coding patient information and specimen.

A cross-sectional study design was conducted on paraffin-embedded lymph node biopsy sections in Jimma University Specialized Hospital (JUSH), the Jimma, Ethiopia. This study was done to determine the magnitude of *M. tuberculosis* among patients suspected for *Tuberculosis lymphadenitis* from December, 2009, to October, 2010. Jimma University is found in Jimma zone, Jimma town Woreda; the town is located 335 km southwest of the capital Addis Ababa. The town has a characteristic of tropical high-land climate condition, heavy rain fall, warm temperature, and long wet period [17].

All suspected patients with clinical presentation of unilateral or bilateral, solitary or matted, painless mass in the regional lymphatic area were included.

Sociodemographic and source of specimen data were collected by predesigned questionnaire. The result was noted on data collection format.

The fresh specimen was immersed in 10% formalin before subjecting the specimen to tissue-processing machine (Leica TP1020). The fixed specimen is then placed in a machine that automatically goes through an elaborate overnight cycle that removes all the water from the specimen and replaces it with paraffin wax.

The next morning the paraffin-impregnated specimen was embedded in a larger block of molten paraffin. Then the block was trimmed and sectioned by microtome (Leica RM2245). Finally, the delicate sections were floated out on a water bath and picked up on a glass slide. The paraffin was dissolved from the tissue on the slide by incubator and stained by H/E and AFB stain separately.

## 3. Result

In this study a total of 60 study participants were included. Of these 20 (33.3%) were males and the rest 40 (66.7%) were females. The female-to-male ratio of study subjects was 2 : 1.

Regarding study participants, age distribution, 14 (23.3%) of the study subjects were below 15 years, 34 (56.7%) within 15–45 years, and 12 (20%) above 45 years (Table 1).

Concerning the site from which the lymph node biopsy is collected, 30 (50%) were collected from cervical, 18 (30%) axillary, 10 (16.7%) mesenteric, and 2 (3.3%) inguinal site. This showed that cervical lymph nodes are more involved than others (Table 1).

**Figure 1:**
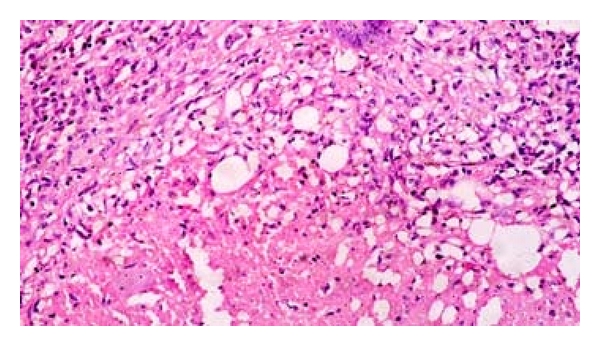
Lymph node biopsy showing epitheloid cell changes in H/E stain by 400x.

Histopathology examination of the specimens revealed the presence of granulomas in the lymph node tissues with marked epitheloid cell changes as well as giant cells formation, but, for the caseation and necrosis, they were present in 51 (85%) cases of nodal biopsies. From those majority, 34 (56.7%) were seen from female samples (Figure 1).

Histopathologically, the granulomas of tuberculosis tend to contain caseation necrosis, but nonnecrotizing granulomas were also been present. The caseating necrosis was present in 51 (85%) of the nodal specimens. Necrotizing granulomas are characteristic of infectious diseases such as tuberculosis and fungal infections as well as rheumatoid nodules, Wegener's granulomatosis, necrobiotic postsurgical granulomas, and others.

**Figure 2:**
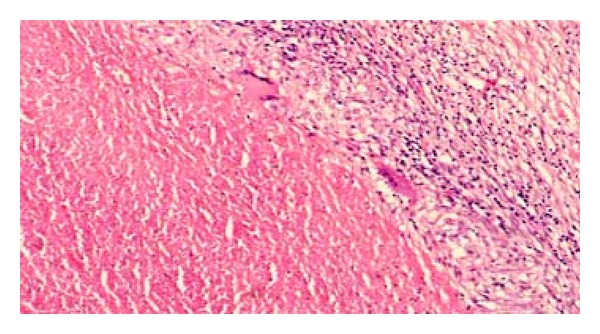
Lymph node biopsy showing giant cells and caseation in H/E stain by 400x.

As it is seen from Figure 2, the presence of visible cheese-like appearance might be associated with necrotizing granulomas as well as necrotic neoplasms. However, non-necrotizing granulomas may occur along with necrotizing granulomas in infectious diseases such as tuberculosis.

**Figure 3:**
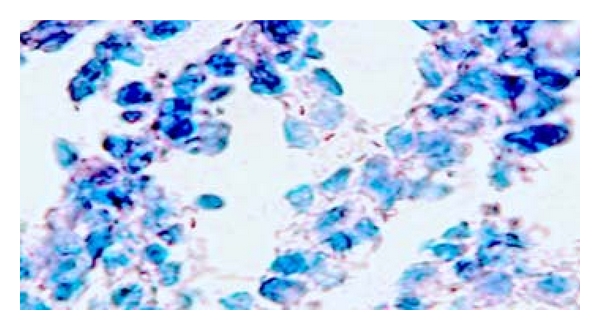
Fragmented or beaded rods (AFB) seen inside and outside macrophages in ZN staining by 400x.

From the total sixty specimens examined by ZN staining technique, AFB was microscopically recognized and confirmed in 37 (61.7%) of the nodal tissue biopsies; the bacilli were scanty in the tissues and were seen in the granulomas associated mainly within the epitheloid cells as well as in the necrotic caseation areas. The AFB seen was fragmented or beaded rods present inside the cells or outside near the cell (macrophages) (Figure 3).

The prevalence of *M. tuberculosis* among the study subjects was 61.7%. The highest percentage of AFB was seen in females (81%), and the rest 19% was in males.

## 4. Discussion

The main limitations of this study is unable to use more sensitive advanced molecular techniques like PCR and culturing method for the detection of *M. tuberculosis. *This is due to facility and logistics constraints. Even if we used less sensitive detection technique, the magnitude of *M. tuberculosis *from lymph node paraffin sections was still high on patients clinically suspected for tuberculosis lymphadenitis at the Jimma University Specialized Hospital. Apart from this, it was impossible to access the HIV status and CD4 count of the study participants because of the high confidentiality of HIV screening result and expensiveness of CD4 count.

In this cross-sectional study sixty study subjects were involved, and lymph node specimens were collected from all individuals from cervical, axillary, mesenteric, and inguinal. All the specimens were stained using both H/E and ZN staining techniques.

After the specimens are stained by H/E staining method, caseating necrosis was observed during microscopic examination in 51 (85%) of the specimens. The presence of caseating necrosis might be due to *M. tuberculosis* or other microorganisms.

The presences of *M. tuberculosis* in 37 lymph node samples were demonstrated using ZN staining technique, and the sources of the lymph node samples were in different sites. As ZN method is relatively confirmatory method than H/E for *M. tuberculosis *from lymph node specimens, the overall prevalence of *M*. *tuberculosis* using ZN staining method was 61.7%.

Cytological diagnosis conducted in enlarged lymph nodes lesions in Russia from a total of 114 patients, 18.5% were positive for tuberculosis, and TBLN is a frequent finding among adult women in two-third of all tuberculosis cases and in children it was reported to be (3.7%) (Table 2) [19].

On the other hand, in our study from a total of 60 analyzed lymph node samples, 40 (66.7%) were from females and 20 (33.3%) were from male patients; from these 37 (61.7%) were positive for AFB by ZN staining technique. From the total examined lymph node tissue sample, most lymph node samples were taken from females and 30 (81%) of female lymph node revealed the presence of AFB. On the other hand, only 7 (18.9%) of males revealed the presence of AFB. This finding is quite different from the gender breakdown seen in pulmonary tuberculosis. For instance a study done in one district of Jimma zone (our catchment area) indicated that majority (65.6%) of the smear positive pulmonary tuberculosis participants were males [23]. From the total positive lymph nodes seen, 30 (81%) were between the age of 15 and 45 years, 7 (18.9%) were under 15 years, and in the age of above 45 years there were no patients with AFB.

A study conducted on a total of 16 patients treated at medical outpatient clinic because of TBLN in Germany; 10% patients has showed cervical lymph nodes, and other localization were supraclavicular, inguinal, and axillary of which 36% showed AFB (Table 2) [19]. Whereas this study revealed that 30 (50%) cervical, 18 (30%) axillary, 10 (16.7%) mesenteric, and 2 (3.3%) inguinal lymph node samples, and from these 37 (61.7%) were positive for AFB by ZN staining.

Another study conducted in Israel for diagnosis and treatment of cervical TBLN, from a total of 21 patients, 18 (85.7%) of them were positive for AFB (Table 2) [20] whereas in our study, from a total of 60 subjects, 37 of them were positive for AFB. The difference between the magnitudes of the two studies might be due to variation in the type of specimen collected.

According to a study done on histopathology of lymph nodal tuberculosis at the Department of Pathology, University of Malaya, 59 cases of tuberculosis cervical lymphadenitis were analyzed by ZN stain and 29 (49.2%) case showed AFB (Table 2) [21].

In a study conducted at North Germany to assess TBLN, cervical lymph node was the most frequently involved (63.3%) followed by mediastinal lymph node (26.7%) and the axillary lymph nodes (8.3%) [24] whereas in this study the most frequently involved lymph node site was cervical (64.9%), axillary (27%), and mediastinal (8%).

A study conducted in the identification of the causative agent of TBLN in rural health center in South Ethiopia revealed the presence of tuberculosis in 75% of patients, whereas in our case the prevalence rate was 61.7%. The gap for the variation in the prevalence might be due to the type of methods used [25].

A study conducted in Butajira, rural Ethiopia, to diagnose TBLN, among a total of 147 clinical suspected patients 72% of them, were confirmed as TBLN (Table 2) [22]. On the other hand, our study has revealed 61.7% and these finding are comparable with each other.

## Figures and Tables

**Table 1:** Sex distribution of Ziehl-Neelsen-stained paraffin-embedded lymph node biopsy sections by anatomical site and age, Jimma University specialized Hospital, the Jimma, Ethiopia, 2010.

Anatomical sites	Male *n* (%)	Female *n* (%)	Total *n* (%)
Cervical Positive Negative Total	3 (10) 3 (10) 6 (20)	19 (63.3) 5 (16.7) 24 (80)	22 (73.3) 8 (26.7) 30 (100)

Axillary Positive Negative Total	3 (16.7) 4 (22.2) 7 (38.9)	9 (50) 2 (11.1) 11 (61.1)	12 (66.7) 6 (33.3) 18 (100)

Mesenteric Positive Negative Total	1 (10) 5 (50) 6 (60)	2 (20) 2 (20) 4 (40)	3 (30) 7 (70) 10 (100)

Inguinal Positive Negative Total	0 1 1	0 1 1	0 2 2

Age distribution			

<15 years Positive Negative Total	1 (7.1) 1 (7.1) 2 (14.2)	6 (42.9) 6 (42.9) 12 (85.8)	7 (50) 7 (50) 14 (100)

15–45 years Positive Negative Total	6 (17.6) 4 (11.8) 10 (29.4)	24 (70.5) 0 24 (70.5)	30 (88.1) 4 (11.8) 34 (100)

>45 years Positive Negative Total	0 8 (66.7) 8 (33.3)	0 4 (33.3) 4 (33.3)	0 12 (100) 12 (100)

**Table 2:** Summery of *M. tuberculosis* prevalence from paraffin-embedded biopsy sections in different countries.

Country (year)	No. of specimens	AFB result
Russia (2002) [18]	114	18.5%
Germany (1999) [19]	16	36%
Israel (2000) [20]	21	85.7%
University of Malaya (1994) [21]	59	49%
Ethiopia (Butajira) (2002) [22]	147	72%
Jimma (this study)	60	61.7%
